# Risen Alive: The Lazarus Phenomenon

**DOI:** 10.1155/2022/3322056

**Published:** 2022-02-15

**Authors:** Waqar Haider Gaba, Shahad Abobakar El Hag, Shaima Mustafa Bashir

**Affiliations:** Internal Medicine Department, Sheikh Khalifa Medical City, Abu Dhabi, UAE

## Abstract

The Lazarus phenomenon described as delayed return of spontaneous circulation (ROSC) after cessation of CPR is rare, though underreported. We present the case of a 25-year-old woman who visited our hospital for persistent vomiting and weight loss for the last six months following bariatric surgery. On the 16^th^ day of admission, the patient experienced cardiac arrest (code blue). The patient underwent 73 min of continuous cardiopulmonary resuscitation (CPR); however, no responses were observed, which led to an announcement of death. Fifty minutes later, the family members noticed subtle eye movements that necessitated resumption of the advanced cardiac life support protocol and resuscitation. The patient survived; however, she developed significant neurological deficits secondary to prolonged anoxic brain injury. She was discharged after a ten-week stay in the hospital but did not achieve full neurologic, cognitive, and motor recovery. Patients should be observed and monitored after the cessation of CPR before confirming death.

## 1. Introduction

The “Lazarus Phenomenon,” also known as autoresuscitation, has been described in patients who achieve spontaneous return of circulation (ROSC) after pronouncement of death following cardiopulmonary resuscitation (CPR). This phenomenon was named after the biblical figure who was resurrected by Jesus 4 days after his death [1]. It was first reported in medical literature in 1982 by Linko et al. [2], and the term Lazarus was used by Bray in 1993 [3]. This phenomenon implies returning to life after being pronounced dead.

Globally, the number of reported cases of the Lazarus phenomenon does not exceed 100. Until now, literature reviews have only been conducted by Adhiyaman et al. [1] (38 cases) and Gordon et al. [4] (65 cases), with less than one-third cases reported to survive.

Varying durations between the time of clinical death and time of ROSC have been reported in previous case reports, although among those who survived, our case had the longest duration of CPR and interval between pronouncement of death and the moment of perception of life confirming ROSC.

Gerard et al. [5] reported that almost 50% of French emergency physicians have encountered autoresuscitation in clinical practice. Moreover, Dhanani et al. [6] stated that 37% of Canadian intensivists have seen at least one case of autoresuscitation in their clinical practice; however, the true incidence remains unknown.

Understanding death is essential to fulfilling a physician's goal of preserving human life. Death is defined as “Death occurs when there is permanent loss of capacity for consciousness and loss of all brainstem functions. This may result from permanent cessation of circulation or catastrophic brain injury. In the context of death determination, ‘permanent' refers to loss of function that cannot resume spontaneously and will not be restored through intervention.” [7] International Liaison Committee on Resuscitation (ILCOR) tried to merge “brain death” and “circulatory death” into one single end point of death. Circulatory death was defined as the absence of circulation evident by the absence of central pulse on palpation, heart sounds on auscultation, breathing, and pupillary response to light. In addition, the following tests should be performed though not mandatory:

1. Asystole or pulseless electrical activity on a continuous ECG display
2. Absence of pulsatile flow during intra-arterial pressure monitoring
3. Absence of contractile activity using echocardiography

Similarly, neurological death was defined clinically as coma, absence of brainstem reflexes, and apnea. Confirmatory tests were suggested though choice of the test depends on the context, pathology, and resources available [8].

Recently, a systematic review was conducted by Advanced Life Support Task Force at ILCOR to look at the feasibility to identify reversible causes of cardiac arrest with Point-Of-Care Ultrasound (POCUS), but the current literature is heterogeneous with high risks of bias, wide confidence intervals, and very low certainty of evidence, which render these data difficult to interpret [9].

Therefore, through this report, we aim to increase awareness of delayed ROSC and the importance of monitoring after the cessation of CPR among healthcare professionals.

## 2. Case Presentation

A 25-year-old woman presented with recurrent intractable vomiting. Her symptoms started 6 months prior to admission at 1 month postbariatric surgery in a private clinic. She had an uneventful surgery with no immediate postoperative complications. She experienced vomiting once or twice daily associated with epigastric pain that worsened after meals. She vomited small amounts of bilious vomit without hematemesis. Additionally, she experienced weakness, fatigue, reduced mobility, and pins-and-needles sensations in her limbs. She had morbid obesity and weighed 115 kg with a body mass index of 41 kg/m^2^ prior to her bariatric surgery. She had no other significant past medical history. She presented to the private clinic emergency department multiple times due to recurrent vomiting. She was given antiemetics and was discharged without needing any hospital admission. She had transient relief but her symptoms persisted. She was a nonsmoker and nondrinker, with no history of diabetes or hypertension. Furthermore, she had no family history of premature cardiac death. However, her father had Takotsubo syndrome with a history of sudden cardiac arrest with good neurological recovery and implantable cardioverter defibrillator (ICD) insertion.

In ED, she was found to be vitally and hemodynamically stable; her physical examinations including cardiac, neurological, and abdominal exams were normal except for bilateral lower limb edema. Blood tests were significant for hypokalemia, acute kidney injury, hypoalbuminemia, deranged liver enzymes, mild coagulopathy, mild microcytic anemia,,, and metabolic acidosis. Lab results on admission are presented in Table 1.

Computed tomography (CT) of the abdomen and pelvis was done in the emergency department, and it ruled out bowel obstruction and thrombosis. In view of the above findings, she was admitted under the care of internal medicine with the initial impression of malnutrition, AKI with hypokalemia, and hepatic derangement for further assessment and management. Ultrasound of the abdomen and liver was reported normal except for features of steatohepatitis. She was reviewed by the gastroenterology team who had the impression of nonalcoholic steatohepatitis likely secondary to rapid weight loss in the last six months. They also advised for esophagogastroduodenoscopy, which showed a postanastomotic ulcer and gastritis. She was hence started on intravenous proton pump inhibitors along with electrolyte replacement and enoxaparin (40 mg SC daily) for venous thromboembolism prophylaxis. Oral intake was encouraged under strict supervision by a dietitian as the patient was at high risk for refeeding syndrome.

Despite those measures, the patient continued to vomit persistently; therefore, she was started on total parenteral nutrition with close monitoring. However, unfortunately, she developed refeeding syndrome with severe electrolyte imbalances, which were managed by supplementing electrolytes. With this deterioration of her condition, a bariatric surgery team was consulted for possible reversal of the gastric bypass, yet they advised for close monitoring and conservative management. During her stay, she developed simple cystitis for which she was treated with oral ciprofloxacin for total of 7 days.

### 2.1. Event History and Timeline

On day 14 of her hospital stay, the patient complained of insomnia and was prescribed 3.75 mg of zopiclone by the on-call physician to help her sleep better at 11:00 pm.

At 02:00 am, nurses noted bradycardia with a heart rate of 48–51 bpm. The patient was examined and found to be asleep but arousable with a heart rate fluctuating from 44 to 48 bpm, oxygen saturation of 95%, and a respiratory rate of 18 breaths/min. Supplemental oxygen was initiated via a nasal cannula at 2 L/min, and the patient was closely monitored. At 02:10 am, the patient was drowsy with a pulse rate of 43 bpm and respiratory rate of 16 breaths/min, and the initial request for the on-call team was initiated. The team arrived at 02:12 am to assess the patient, but at 02:15 am, the patient's Glasgow Coma Scale (GCS) score decreased to 3, and a code blue was announced. Basic life support (BLS) was initiated by ward staff, followed by advanced cardiac life support (ACLS) on the arrival of the code response team (Appendix 1. Represents the Utstein report-CPR sheet). The initial rhythm was asystole. After a few cycles, the rhythm changed to refractory ventricular fibrillation, followed by pulseless ventricular tachycardia, which was treated with defibrillation; amiodarone (IV) and lidocaine (IV) were administered as per protocol. The rhythm changed to pulseless electrical activity (PEA), which was managed with CPR compressions, epinephrine (IV) administration every 3–5 min, and other measures as per protocol. The patient was immediately intubated. According to the documentation, the patient underwent CPR for 73 min, with 23 doses of intravenous (IV) epinephrine, sodium bicarbonate (50 mg IV administered twice), magnesium sulphate (2 g IV administered once), amiodarone (150 mg IV administered once), calcium gluconate (1 g IV administered once), and lidocaine (20 mg/5 mL IV administered once); direct current shock was repeated thrice. The responding code team administered 50 mg of IV alteplase push twice due to suspicions of a massive pulmonary embolism.

Unfortunately, not sufficient dose of amiodarone was given and torsade was not initially considered secondary to zopiclone especially in the presence of hypokalemia.

Her pulse was assessed manually and confirmed by ultrasound. Doppler ultrasound showed the absence of left ventricular contractions and carotid artery pulse and flow. The monitor displayed PEA. Bedside echocardiography was performed during uninterrupted CPR, which showed no evidence of cardiac tamponade but showed flickering of the myocardium with the absence of valve opening. The pupils were fully dilated and nonreactive; no audible heart sounds, a nonpalpable pulse, and cold skin indicating lack of perfusion were noted. Both bedside Doppler and echocardiography were repeated several times before stopping resuscitative efforts. CPR was discontinued at 03:28 am, with the monitor showing PEA. Based on PEA and the absence of cardiac movements on echocardiography, death was pronounced at that time, and the family was informed.

Unfortunately, there was no evidence of any monitoring including cardiac monitoring after pronouncement of death. Family was left in the room without any monitors while waiting for death certificate and transfer to mortuary as per hospital protocol.

**Figure 1:**
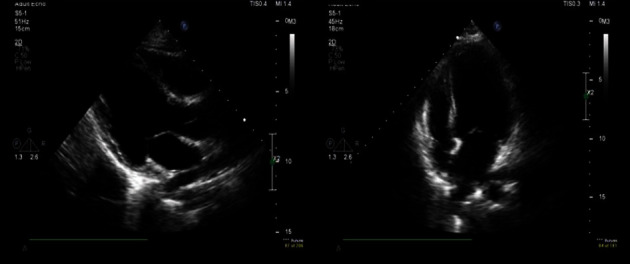
Echocardiogram postcardiac arrest and resurrection. Apical view and long axis parasternal view.

At 04:18 am, the patient's family noticed movement in the patient's eyes. An intensive care unit (ICU) doctor examined the patient, and another code blue was announced at 04:19 am. Upon examination, both pupils were reactive to light. Faint pulses in the carotid area and contractions of the heart and carotid arteries were also observed on ultrasound. ROSC was announced at 04:25 am, and the patient was stable at 04:45 am. The patient was pulseless for approximately 123 min. The cardiology team performed bedside echocardiography, which showed severe left ventricular dysfunction, global dyskinesia, normal right ventricular size, and no pericardial effusion (Figure 1).

An extensive blood workup was carried out including complete blood count, comprehensive metabolic panel, lactic acid, troponin, coagulation panel, and blood gas. The laboratory tests done almost 24 hours prior to the arrest were also reviewed and are represented in Table 2.

The laboratory findings at 04:46 am (after cardiac arrest) are presented in Table 3.

**Figure 2:**
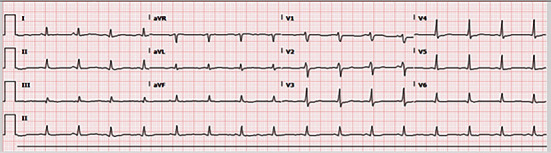
Precardiac arrest ECG.

**Figure 3:**
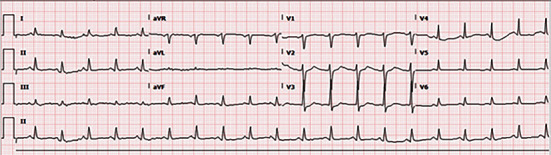
Postcardiac arrest ECG.

An ECG done the night before showed sinus rhythm with QTc interval of 462 (Figure 2). The one done at 6:50 am post the event showed sinus tachycardia of 112 bpm and a QTc interval of 432 with no ischemic changes (Figure 3).

The patient was started on IV hydration with 0.9% normal saline at 70 mL/h, IV heparin infusion, IV fentanyl infusion, and midazolam infusion as per hospital protocol. The patient was referred to the interventional radiology department for emergency thrombectomy at 05:00 am. A pulmonary angiogram showed patent bilateral pulmonary artery branches without significant central thromboembolic disease, and her pulmonary artery pressure was 30/20 mmHg (mean: 23 mmHg). The patient was transferred to the ICU afterward.

### 2.2. Differential Diagnoses

Lazarus phenomenon, massive pulmonary embolism, cardiac arrhythmias.

### 2.3. Case Progress

In the ICU, 10 hours later, the patient developed hypotension (blood pressure: 70/40 mmHg). A bedside ultrasound scan showed a large amount of free fluid in the abdomen, and repeat laboratory examinations revealed decreased hemoglobin level (34 g/L), international normalized ratio > 10.0, D-dimer level > 20 mcg/mL, platelet count (129 × 10^9^/L), and fibrinogen level < 0.40 g/L. Considering severe coagulopathy, the patient's status postcardiac arrest, and recent thrombolysis with alteplase, a diagnosis of acute intra-abdominal bleed was made, which was managed according to the massive transfusion protocol. The patient was taken to the operating room for emergency laparotomy. During the operation, bleeding was noted from the lesser sac (gastrohepatic ligament) behind the caudate lobe of the liver, but no bleeding source was identified, and bleeding stopped with correction of the severe coagulopathy; hence, the surgery team decided to proceed with packing. On the day of the operation, she developed anuric acute kidney injury. Nephrology team was consulted, and she was initiated on furosemide infusion at 5 mg/hr for a total of 7 days before a notable improvement of renal function and urine output was appreciated. Her kidney function continued to improve until full recovery was achieved in the next 2 weeks. The next day, she was taken to theatres for relook when packs were removed and repacking was done with no active bleeding noted. Three days later, packs were removed with insertion of gastrostomy tube and mesh closure of abdomen. One week later, she had another massive bleed on the background of severe coagulopathy and malnutrition. This time she has superficial bleed around the mesh placed. There was no intraperitoneal bleeding. Mesh was removed and packing was done again. Her case was addressed in a multidisciplinary approach including ICU, GI, hematology, and general surgery. Correction of coagulopathy was done during these repeated laparotomies. GI imaging and GI Endoscopy failed to identify any source of bleeding.

Furthermore, during her ICU stay, she was diagnosed with candidemia and a urinary tract infection caused by *Escherichia coli*, which were managed as per antibiotic guidelines and protocol of the hospital. She was successfully extubated on day 22 of her ICU stay. She spent a total of 41 days in the ICU after which she was transferred to the Medical High Dependency Unit (MHDU) under the care of internal medicine. Post-ICU discharge, she developed myoclonus with motor and sensory axonal neuropathy, as seen on nerve conduction studies and electromyography. Considering her presentation post-ICU discharge, a diagnosis of mini-poly-myoclonus secondary to anoxic brain injury was made by the neurology team.

### 2.4. Outcome and Follow-Up

Care was transferred to the rehabilitation team 11 days after the patient was transferred to the MHDU. A plan was formulated for extensive physiotherapy for 4 weeks owing to residual generalized severe rigidity. She achieved mobility with a walker and was able to ascend and descend stairs. After a complicated hospital course and extensive rehabilitation, she was able to leave the hospital with plans for outpatient rehabilitation. Unfortunately, she was readmitted to the ICU at 13 days after discharge due to jaundice, confusion, agitation, abdominal distension, and a GCS score of 8. She was diagnosed with acute ischemic hepatic injury and referred to a liver transplant center.

Her anemia was thought to be multifactorial including recent thrombolytic therapy, severe malnutrition, and severe coagulopathy. Her overall condition was thought to be due to a combination of existing non-alcoholic steatohepatitis, recent prolonged CPR and resuscitation, repeated hemodynamic instability, severe coagulopathy, and underlying severe malnutrition leading to compromised hepatic function and ischemia.

## 3. Discussion

Our case report of a 25-year-old female who experienced ROSC at 50 min after the pronouncement of death presents a rare occurrence called autoresuscitation or the Lazarus phenomenon. Although such occurrence is rare, it is often underreported [1].

In our case, the absence of monitoring during the 50-minute period between cessation of CPR and perception of signs of life observed by family members raise the possibility that duration might have been shorter and highlights it as limitations of our study.

Several mechanisms may be involved in the development of the Lazarus phenomenon, including autopositive end-expiratory pressure (PEEP) in ventilated patients, severe electrolyte imbalances (particularly hyperkalemia and hypokalemia), reversible causes during CPR, and sufficient time allowance for CPR to achieve ROSC.

Our patient's cardiac arrest and death may be due to several reasons. Considering her history of bariatric surgery and a sedentary lifestyle, we considered pulmonary embolism as one of the causes, which was managed effectively with thrombolysis during CPR. Additionally, arrhythmia and cardiac arrest induced by electrolyte imbalances were considered. These were evidenced by bradyarrhythmia and abnormal electrolytes, which were addressed during CPR. Another cause may be a preexisting cardiac condition evidenced by the cardiac arrest experienced by her father, which required an ICD. Furthermore, medication history in this case is very important. This includes that fact that the patient was on ciprofloxacin for UTI treatment, and she received zopiclone the night of the event. Both medications are associated with QT prolongation, which was already seen on ECG prior to her arrest. The fact that she received those medications in the setting of hypokalemia and prolonged QT interval puts her at a significant risk of arrhythmias including torsade de pointes.

Adhiyaman et al. [1] investigated the causes of arrest and outcomes of 38 patients. They found that the mean duration of CPR was 27 min and the mean time for spontaneous recovery was 10 min. Seventeen patients (45%) achieved good neurological recovery following ROSC. Among them, 3 subsequently died during their hospital stay due to sepsis and pulmonary embolism. The remaining 14 patients (35%) were eventually discharged home with no significant neurological sequelae. Seventeen patients (45%) were unable to achieve neurological recovery following ROSC and died soon after. The outcome was not known in 4 patients (10%). However, no significant correlation was found between the outcome, CPR duration, and time interval for ROSC or diagnosis [1]. Gordon et al. [4] reported 65 patients with ROSC after termination of resuscitation, 18 of which (28%) made a full recovery.

Here, we discuss different cases reported in the literature regarding the Lazarus phenomenon with the possible pathogenesis, CPR duration, the time of achieving autoresuscitation, and outcomes. In some cases, electromechanical dissociation may be a contributory factor. Intraoperative arrest was reported in a patient with a cardiac pacemaker, which may be due to auto-PEEP since the patient's condition improved after being disconnected from the ventilator [10]. Auto-PEEP may be recognized as a cause of reversible death in ventilated ICU patients through the measurement of end tidal CO_2_, especially in patients who experience PEA for <1 min after being disconnected from the ventilator. Two case reports discussed occult auto-PEEP as death induced by cardiac arrest with possible reversal after ventilator disconnection [11, 12]. In a patient with inferior myocardial infarction, acute coronary syndrome was the reported cause of reversible death due to cardiac arrest. In this case, the patient was pronounced clinically dead after 35 min of CPR; however, he was found moving in the mortuary at 20 min after being pronounced dead but died 4 days later [13].

The length of the observation period may affect the recognition of the Lazarus phenomenon. Previous studies have provided diverse recommendations regarding the time of observation. Sheth et al. [14] investigated the sufficiency of a 2-min observation period after asystole before declaring the death of a patient, and no cases of autoresuscitation were seen in the 73 patients after 2 min of asystole. Furthermore, Dhanani et al. [15] found that circulation did not resume after 89 s of absence in 41 ICU patients who decided to withdraw from life-sustaining therapy. One case report described the spontaneous return of cardiac rhythm after observation for at least 10 min after stopping CPR [16]. Another case report of hyperkalemia-induced cardiac arrest found that the patient responded to sodium bicarbonate at 7 min after the cessation of 35 min of CPR [17]. Similarly, a patient with chronic kidney disease and hyperkalemia-induced cardiac arrest recovered 8–10 min after the cessation of 26 min of CPR [18]. These reports were consistent with another report, which recommended continuous monitoring of patients for at least 10 min after the cessation of CPR [10].

Differences in duration for which CPR was performed were also noted in previous studies. Skulberg reported spontaneous circulation after the cessation of resuscitation in 5 patients who all had asystole, which was diagnosed with electrocardiography by anesthesiologists [19]. After 30 min, the medical staff decided to discontinue resuscitative efforts. Among the 5 patients, 2 were discharged but 3 died after a few hours. Among the discharged patients, one developed dementia while the other had no cerebral sequelae [19]. Considering these findings, the inclusion of the Lazarus phenomenon in the ACLS and BLS resuscitation guidelines is reasonable and may improve patient outcomes.

Few case reports have demonstrated patients who achieved complete recovery with no neurologic sequelae. A female patient recovered after 4 min of asystole post-complete heart block and failed temporary transcutaneous pacing due to a “Do Not Resuscitate” order. The patient was placed on palliative sedation; however, she regained consciousness and spontaneous junctional rhythm with full neurological recovery [20]. A 68-year-old woman developed sudden cardiac arrest secondary to hyperkalemia with renal insufficiency. Despite 100 min of CPR and hyperkalemia treatment, the cardiac arrest persisted. Hemodialysis was then initiated during CPR; after 20 min, she regained a spontaneous heartbeat. No neurologic sequelae were observed after her recovery [21]. Another male patient experienced a drug overdose and required high doses of naloxone. He regained consciousness but experienced cardiac arrest during transit to the hospital. He achieved ROSC at 1 min after the cessation of 25 min of CPR with full neurological recovery; he was discharged after a complicated ICU course of 18 days [22].

For some patients, the outcome was fatal. A 94-year-old patient experienced cardiac arrest due to massive hemorrhage during duodenal perforation repair. The patient gained spontaneous rhythm at 2–3 min after the cessation of 40 min of CPR due to electromechanical dissociation. The patient showed improvement for 72 h but eventually died within 18 days of hospitalization due to septic shock and multi-organ failure [23]. Hanning et al. [24] reported a 67-year-old man with an out-of-hospital cardiac arrest and prolonged CPR for nearly 1 h. Once resuscitation efforts were stopped, he had a faint central pulse 5 min later and regained consciousness 1 h later. He died 22 h after the initial cardiac arrest. A systematic review by Ballesteros-Peña et al. [25] found that 64% of all patients died before discharge. Additionally, there is a risk of neurological impairment even when a patient survives. This may be due to the delayed effects of the medications administered during CPR; when CPR is ceased, venous return improves, increasing the delivery of these drugs, including adrenaline [25]. In general, prognosis after autoresuscitation is poor.

## 4. Conclusion

The Lazarus phenomenon is underreported and underrecognized, even among health professionals. Our findings confirm that resuscitation efforts including CPR should not be terminated until a treatable cause is present. Furthermore, once resuscitation is terminated, patients should be observed closely with cardiac monitoring and electrocardiography before pronouncing death. Moreover, the inclusion of information regarding the Lazarus phenomenon in future ACLS guidelines should be considered.

### 4.1. Patient Perspective

Our patient made a remarkable recovery with the assistance of the rehabilitation team. She thanked the entire medical team including the ICU and surgical team. She was so excited to be discharged; unfortunately, she was readmitted and transferred to a liver transplant center.

## Figures and Tables

**Table 1:** Lab results on admission day.

Blood test	Result (normal range)
Potassium	2.8 mmol/L (3.5–5.0 mmol/L)
Magnesium	0.80 mmol/L (0.66–1.07 mmol/L)
Corrected calcium	2.35 mmol/L (2.15–2.55 mmol/L)
Phosphorus	1.03 mmol/L (0.81–1.45 mmol/L)
HCO_3_	18 mmol/L (22–28 mmol/L)
Creatinine	140 micromol/L (44–80 mmol/L)
eGFR	45 ml/min (>60 mL/min)
Albumin	17 g/L (35–52 g/L)
AST	180 IU/L (<32 IU/L)
ALT	121 IU/L (<33 IU/L)
ALK phosphatase	180 IU/L (35–104 IU/L)
Bilirubin Total	21.2 micromol/L (<21 micomol/L)
Bilirubin direct	19.4 micromol/L (<5 micomol/L)
INR	1.5 (0.7–1.1)
PT	17.4 sec (<14 sec)
CRP	11.1 mg/L (<5 mg/L)
Hemoglobin	108 g/L (117–161 g/L)
MCV	72 fL (81–108 fL)

**Table 2:** Lab results 24 hours prior to the event.

Blood test	Result (normal range)
Potassium	3.3 mmol/L (3.5–5.0 mmol/L)
HCO_3_	22 mmol/L (22–28 mmol/L)
Creatinine	26 micromol/L (44–80 mmol/L)
Albumin	27 g/L (35–52 g/L)
AST	69 IU/L (<32 IU/L)
ALT	35 IU/L (<33 IU/L)
Phosphorus	0.58 mmol/L (0.66–1.07 mmol/L)
Hemoglobin	86 g/L (117–161 g/L)
MCV	72 fL (81–108 fL)

**Table 3:** Post ROSC—04:46 am.

Blood test	Result (normal range)
Potassium	2.4 mmol/L (3.5–5.0 mmol/L)
pH	7.19 (7.35–7.45)
HCO_3_	10 mmol/L (22–28 mmol/L)
Creatinine	38 micromol/L (44–80 mmol/L)
Lactic acid	12.1 mmol/L (<2.2 mmol/L)
Troponin	25 mmol/L (<14 mmol/L)
WBC	22.0 × 10^9^ g/L (4.4–11.1 × 10^9^ g/L)
INR	3.5 (0.7–1.1)
PT	38.8 sec (<14 sec)
Hemoglobin	96 g/L (117–161 g/L)

## Data Availability

The data used to support the findings of this case study are included within the article.

## Supplementary Materials

Supplementary Materials Appendix 1: representing the Utstein report-CPR sheet.
