# Supplementary material for: Risen Alive: The Lazarus Phenomenon
Source: Case Rep Crit Care. 2022 Feb 15;2022:3322056. doi: 10.1155/2022/3322056 (PMC8863492; doi:10.1155/2022/3322056)
Supplement: Supplementary Materials — Appendix 1: representing the Utstein report-CPR sheet. [file 3322056.f1.pdf]

[redacted]

Cardiopulmonary Resuscitation Report

[redacted]

Date: [redacted] | Time Event Recognized 2:15 | Location: [redacted] | Witnessed: ☑ Yes ☐ No
Code ☑ Blue ☐ Pink | Team Arrival Time: 2:16 | Weight (Kg): ____ | Height (cm): ____

Hospital-wide resuscitation response activated? ☐ Yes ☐ No | Was patient conscious at onset? ☐ Yes ☐ No

Condition when need for compressions/defibrillation was identified? ☐ Pulseless ☐ Pulse (poor perfusion)

Did the patient with a pulse requiring compressions become pulseless? ☐ Yes ☐ No

Airway/ Ventilation

Breathing	Ventilation	Intubation	Confirmation
☐ Spontaneous	☐ Bag-Valve-Mask	Time: 2:20	☐ Auscultation
☐ Apneic	☑ Endotracheal Tube	Size : ____	☐ Exhaled CO3
☐ Agonal	☐ Tracheostomy	By Whom:	☐ Other ____
☑ Assisted	☐ Other: ____		

Circulation

1st Rhythm Requiring Compressions: Asystole
1st Pulseless Rhythm: ____
Time Chest Compressions Started: 2:15
Impedance Threshold Device used? ☐ Yes ☐ No
AED Applied: ☑ Yes ☐ No Time: ____
Pacemaker On: ☐ Yes ☐ No Time: ____
Defibrillator Type(s): ____

Outcome

Time Resuscitation Event Ended: ____

Reason Resuscitation Ended:

☐ Survived – Return of Circulation (ROC) >20 min
☐ Died – Efforts Terminated (No Sustained ROC)

Time	Pulse		HR	Rhythm	BP	Breathing		SpO2	CPR	Defibrillator Type AED / Manual	Joules	Bolus Medications (* if given by ET Tube)						Infusions Dose / cc per hr				Other Infusions /Volume replacement or Blood products given	Comments
	Spontaneous	Assisted				Spontaneous	Assisted					Epinephrine	Atropine	Sodium Bicarbonate	Adenosine	Amiodarone	Lidocaine	Dopamine	Dobutamine	Epinephrine	Norepinephrine		
2:15		✓	Asystole																			NS 500 mL bolus given	
2:17		✓	"									✓											
2:19		✓	"																				
2:20		✓	"									✓											
2:22		✓	"																			D50 50mg given + NS	1L bolus initiated
2:23		✓										✓		✓									
2:24		✓	PEA																				
2:25		✓																					
2:26		✓	PEA									✓											
2:28		✓	"																				
2:29		✓										✓											
2:30		✓	PEA																				
2:32		✓	PEA									✓											

Rev.-June 2014)

[redacted]

Cardiopulmonary Resuscitation Report

33
29

Date: ________	Time Event Recognized ________	Location: ________	Witnessed: ☐ Yes ☐ No
Code ☐ Blue ☐ Pink	Team Arrival Time: ________	Weight (Kg): ________	Height (cm): ________

Hospital-wide resuscitation response activated? ☐ Yes ☐ No | Was patient conscious at onset? ☐ Yes ☐ No

Condition when need for compressions/defibrillation was identified? ☐ Pulseless ☐ Pulse (poor perfusion)

Did the patient with a pulse requiring compressions become pulseless? ☐ Yes ☐ No

Airway/ Ventilation

Breathing	Ventilation	Intubation	Confirmation
☐ Spontaneous	☐ Bag-Valve-Mask	Time: ________	☐ Auscultation
☐ Apneic	☐ Endotracheal Tube	Size: ________	☐ Exhaled CO3
☐ Agonal	☐ Tracheostomy	By Whom:	☐ Other ________
☐ Assisted	☐ Other: ________	________	

Circulation

1st Rhythm Requiring Compressions: ________

1st Pulseless Rhythm: ________

Time Chest Compressions Started: ________

Impedance Threshold Device used? ☐ Yes ☐ No

AED Applied: ☐ Yes ☐ No Time: ________

Pacemaker On: ☐ Yes ☐ No Time: ________

Defibrillator Type(s): ________

Outcome

Time Resuscitation Event Ended: ________

Reason Resuscitation Ended:

☐ Survived – Return of Circulation (ROC) >20 min

☐ Died – Efforts Terminated (No Sustained ROC)

Time	Pulse: Spontaneous	Pulse: Assisted	HR	Rhythm	BP	Breathing: Spontaneous	Breathing: Assisted	SpO2	CPR	Defibrillator Type AED / Manual	Joules	Bolus Medications (* if given by ET Tube): Epinephrine	Atropine	Sodium Bicarbonate	Adenosine	Amiodarone	Lidocaine	Infusions Dose / cc per hr: Dopamine	Dobutamine	Epinephrine	Norepinephrine	Other Infusions /Volume replacement or Blood products given	Comments
2:33		✓	PEA						✓			✓											
2:34		✓	FINE VF						✓	✓ -	150												
2:36		✓	FINE VF						✓	✓ - ✓ -	200 200	✓											
2:38		✓	PEA						✓								✓						
2:39		✓							✓			✓											
2:40		✓	PEA						✓														
2:42		✓	pulseless vtach						✓	✓	200												
2:44		✓	PEA						✓							✓							
2:45		✓							✓			✓											
2:46		✓	PEA						✓													Mg Sulfate 2g given	
2:48		✓	PEA						✓			✓										Alteplase 50mg given	
2:50		✓	PEA						✓														
2:5[illegible]									✓			✓											

HOS 060023(HIMC-Rev. June 2014)

[redacted]

Cardiopulmonary Resuscitation Report

Date: ______	Time Event Recognized ______	Location: ______	Witnessed: ☐ Yes ☐ No
Code ☐ Blue ☐ Pink	Team Arrival Time: ______	Weight (Kg): ______	Height (cm): ______

Hospital-wide resuscitation response activated? ☐ Yes ☐ No | Was patient conscious at onset? ☐ Yes ☐ No

Condition when need for compressions/defibrillation was identified? ☐ Pulseless ☐ Pulse (poor perfusion)

Did the patient with a pulse requiring compressions become pulseless? ☐ Yes ☐ No

Airway/ Ventilation

Breathing	Ventilation	Intubation	Confirmation
☐ Spontaneous	☐ Bag-Valve-Mask	Time: ______	☐ Auscultation
☐ Apneic	☐ Endotracheal Tube	Size: ______	☐ Exhaled CO3
☐ Agonal	☐ Tracheostomy	By Whom:	☐ Other ______
☐ Assisted	☐ Other: ______		

Circulation

1st Rhythm Requiring Compressions: ______
1st Pulseless Rhythm: ______
Time Chest Compressions Started: ______
Impedance Threshold Device used? ☐ Yes ☐ No
AED Applied: ☐ Yes ☐ No Time: ______
Pacemaker On: ☐ Yes ☐ No Time: ______
Defibrillator Type(s): ______

Outcome

Time Resuscitation Event Ended: ______

Reason Resuscitation Ended:

☐ Survived – Return of Circulation (ROC) >20 min
☐ Died – Efforts Terminated (No Sustained ROC)

Time	Pulse: Spontaneous	Pulse: Assisted	HR	Rhythm	BP	Breathing: Spontaneous	Breathing: Assisted	SpO2	CPR	Defibrillator Type AED / Manual	Joules	Bolus Medications (* if given by ET Tube): Epinephrine	Atropine	Sodium Bicarbonate	Adenosine	Amiodarone	Lidocaine	Infusions Dose / cc per hr: Dopamine	Dobutamine	Epinephrine	Norepinephrine	Other Infusions /Volume replacement or Blood products given	Comments
2:52			PEA						✓														
2:54			PEA						✓			✓											
2:56			PEA						✓					✓								Calcium Chloride 1g	
2:57			PEA						✓			✓											
2:58			PEA						✓														
3:00			PEA						✓			✓											
3:02			PEA						✓														
3:03			PEA						✓			✓											
3:04			PEA						✓														
3:05			PEA						✓														
3:06			PEA						✓			✓											
3:07			PEA						✓													Alteplase 50mg given	
3:08			PEA						✓														

Legend

(Rev.-June 2014)

[redacted]

[redacted]

Cardiopulmonary Resuscitation Report

[redacted]

last modified 15-01-2014

Date: ________	Time Event Recognized ________	Location: ________	Witnessed: ☐ Yes ☐ No
Code ☐ Blue ☐ Pink	Team Arrival Time: ________	Weight (Kg): ________	Height (cm): ________

Hospital-wide resuscitation response activated? ☐ Yes ☐ No | Was patient conscious at onset? ☐ Yes ☐ No

Condition when need for compressions/defibrillation was identified? ☐ Pulseless ☐ Pulse (poor perfusion)

Did the patient with a pulse requiring compressions become pulseless? ☐ Yes ☐ No

Airway/ Ventilation

Breathing	Ventilation	Intubation	Confirmation
☐ Spontaneous	☐ Bag-Valve-Mask	Time: ________	☐ Auscultation
☐ Apneic	☐ Endotracheal Tube	Size : ________	☐ Exhaled CO3
☐ Agonal	☐ Tracheostomy	By Whom: ________	☐ Other ________
☐ Assisted	☐ Other: ________		

Circulation

1st Rhythm Requiring Compressions: ________
1st Pulseless Rhythm: ________
Time Chest Compressions Started: ________
Impedance Threshold Device used? ☐ Yes ☐ No
AED Applied: ☐ Yes ☐ No Time: ________
Pacemaker On: ☐ Yes ☐ No Time: ________
Defibrillator Type(s): ________

Outcome

Time Resuscitation Event Ended: ________

Reason Resuscitation Ended:

☐ Survived – Return of Circulation (ROC) >20 min
☐ Died – Efforts Terminated (No Sustained ROC)

Time	Pulse: Spontaneous	Pulse: Assisted	HR	Rhythm	BP	Breathing: Spontaneous	Breathing: Assisted	SpO2	CPR	Defibrillator Type AED / Manual	Joules	Bolus Medications (* if given by ET Tube): Epinephrine	Atropine	Sodium Bicarbonate	Adenosine	Amiodarone	Lidocaine	Infusions Dose / cc per hr: Dopamine	Dobutamine	Epinephrine	Norepinephrine	Other Infusions /Volume replacement or Blood products given	Comments
3:09				PEA					✓			✓											
3:10				PEA					✓														
3:12				PEA					✓			✓											
3:14				PEA					✓														
3:15									✓			✓											
3:16				PEA					✓														
3:18				PEA					✓			✓											
3:20				PEA					✓														
3:21									✓			✓											
3:22				PEA					✓														
3:23									✓			✓											
3:24				PEA					✓														
[illegible]				PEA																			

Legend
[redacted]
hycardia
re:sure
n via ET

[redacted]

Cardiopulmonary Resuscitation Report

[redacted]

:33
:29

last modified 15-01-2014

Date:	Time Event Recognized ______	Location: ______	Witnessed: ☐ Yes ☐ No
Code ☐ Blue ☐ Pink	Team Arrival Time: ______	Weight (Kg): ______	Height (cm): ______

Hospital-wide resuscitation response activated? ☐ Yes ☐ No | Was patient conscious at onset? ☐ Yes ☐ No

Condition when need for compressions/defibrillation was identified? ☐ Pulseless ☐ Pulse (poor perfusion)

Did the patient with a pulse requiring compressions become pulseless? ☐ Yes ☐ No

Airway/ Ventilation

Breathing	Ventilation	Intubation	Confirmation
☐ Spontaneous	☐ Bag-Valve-Mask	Time: ______	☐ Auscultation
☐ Apneic	☐ Endotracheal Tube	Size : ______	☐ Exhaled CO3
☐ Agonal	☐ Tracheostomy	By Whom: ______	☐ Other ______
☐ Assisted	☐ Other: ______		

Circulation

1st Rhythm Requiring Compressions: ______
1st Pulseless Rhythm: ______
Time Chest Compressions Started: ______
Impedance Threshold Device used? ☐ Yes ☐ No
AED Applied: ☐ Yes ☐ No Time: ______
Pacemaker On: ☐ Yes ☐ No Time: ______
Defibrillator Type(s): ______

Outcome

Time Resuscitation Event Ended: ______

Reason Resuscitation Ended:

☐ Survived – Return of Circulation (ROC) >20 min
☐ Died – Efforts Terminated (No Sustained ROC)

Time	Pulse		HR	Rhythm	BP	Breathing		SpO2	CPR	Defibrillator Type AED / Manual	Joules	Bolus Medications (* if given by ET Tube)						Infusions Dose / cc per hr				Other Infusions /Volume replacement or Blood products given	Comments
	Spontaneous	Assisted				Spontaneous	Assisted					Epinephrine	Atropine	Sodium Bicarbonate	Adenosine	Amiodarone	Lidocaine	Dopamine	Dobutamine	Epinephrine	Norepinephrine		
												/											
3:27																						Resuscitation stopped	
3:28						PEA																	
																						* ECHO done - w/ flutter but no cardiac contractions	
																						Ultrasound of the carotid done - no blood flow / pulse	

[redacted]

-Sinus Tachycardia
P-Blood Pressure
ith * if given via ET

HOS 060023(HMC-Rev.-June 2014)

Cardiopulmonary Resuscitation Report

Date: [illegible]	Time Event Recognized 04:19	Location:	Witnessed: ☑ Yes ☐ No
Code ☑ Blue ☐ Pink	Team Arrival Time: 04:19	Weight (Kg):	Height (cm):

Hospital-wide resuscitation response activated? ☐ Yes ☐ No | Was patient conscious at onset? ☐ Yes ☐ No

Condition when need for compressions/defibrillation was identified? ☐ Pulseless ☐ Pulse (poor perfusion)

Did the patient with a pulse requiring compressions become pulseless? ☐ Yes ☐ No

Airway/ Ventilation

Breathing	Ventilation	Intubation	Confirmation
☐ Spontaneous	☐ Bag-Valve-Mask	Time:	☐ Auscultation
☐ Apneic	☑ Endotracheal Tube	Size :	☐ Exhaled CO3
☐ Agonal	☐ Tracheostomy	By Whom:	☐ Other
☑ Assisted	☐ Other:		

Circulation

1st Rhythm Requiring Compressions:
1st Pulseless Rhythm:
Time Chest Compressions Started:
Impedance Threshold Device used? ☐ Yes ☐ No
AED Applied: ☐ Yes ☐ No Time:
Pacemaker On: ☐ Yes ☐ No Time:
Defibrillator Type(s):

Outcome

Time Resuscitation Event Ended:

Reason Resuscitation Ended:

☐ Survived – Return of Circulation (ROC) >20 min
☐ Died – Efforts Terminated (No Sustained ROC)

Time	Pulse: Spontaneous	Pulse: Assisted	HR	Rhythm	BP	Breathing: Spontaneous	Breathing: Assisted	SpO2	CPR	Defibrillator Type AED / Manual	Joules	Bolus Medications (* if given by ET Tube): Epinephrine	Atropine	Sodium Bicarbonate	Adenosine	Amiodarone	Lidocaine	Infusions Dose / cc per hr: Dopamine	Dobutamine	Epinephrine	Norepinephrine	Other Infusions /Volume replacement or Blood products given	Comments
04:19	✓		112	ST	108/90		✓	100%															ī pupil reactive to light
04:24	✓		108	ST	113/63		–	100%														Norepinephrine 0.1mcg started	+ NS 100ml/hr
04:45	✓		118	ST	124/78		✓	100%														Fentanyl 50mcg given	
04:55	✓		113	ST	164/74		✓	100%														Patient reached ROSC	

cardia
sure
a ET

HOS 060023(HIMC-Rev.-June 2014)
